# Experience with use of extracorporeal life support for severe refractory status asthmaticus in children

**DOI:** 10.1186/cc7735

**Published:** 2009-03-02

**Authors:** Kiran B Hebbar, Toni Petrillo-Albarano, Wendy Coto-Puckett, Micheal Heard, Peter T Rycus, James D Fortenberry

**Affiliations:** 1 Department of Pediatrics, Emory University School of Medicine, 1405 Clifton Road, Atlanta, GA 30322, USA; 2 Extracorporeal Life Support Organization (ELSO), University of Michigan, 503 Thompson Street, Ann Arbor MI 48109-1318, USA; 3 Department of Critical Care, Children's Healthcare of Atlanta at Egleston, 1405 Clifton Road, Atlanta, GA 30322, USA

## Abstract

**Introduction:**

Severe status asthmaticus (SA) in children may require intubation and mechanical ventilation with a subsequent increased risk of death. In the patient with SA and refractory hypercapnoeic respiratory failure, use of extracorporeal life support (ECLS) has been anecdotally reported for carbon dioxide removal and respiratory support. We aimed to review the experience of a single paediatric centre with the use of ECLS in children with severe refractory SA, and to compare this with international experience from the Extracorporeal Life Support Organization (ELSO) registry.

**Methods:**

All paediatric patients (aged from 1 to 17 years) with primary International Classification of Diseases (ICD)-9 diagnoses of SA receiving ECLS for respiratory failure from both the Children's Healthcare of Atlanta at Egleston (Children's at Egleston) database and the ELSO registry were reviewed.

**Results:**

Thirteen children received ECLS for refractory SA at the Children's at Egleston from 1986 to 2007. The median age of the children was 10 years (range 1 to 16 years). Patients generally received aggressive use of medical and anaesthetic therapies for SA before cannulation with a median partial pressure of arterial carbon dioxide (PaCO_2_) of 130 mmHg (range 102 to 186 mmHg) and serum pH 6.89 (range 6.75 to 7.03). The median time of ECLS support was 95 hours (range 42 to 395 hours). All 13 children survived without neurological sequelae. An ELSO registry review found 64 children with SA receiving ECLS during the same time period (51 excluding the Children's at Egleston cohort). Median age, pre-ECLS PaCO_2 _and pH were not different in non-Children's ELSO patients. Overall survival was 60 of 64 (94%) children, including all 13 from the Children's at Egleston cohort. Survival was not significantly associated with age, pre-ECLS PaCO_2_, pH, cardiac arrest, mode of cannulation or time on ECLS. Significant neurological complications were noted in 3 of 64 (4%) patients; patients with neurological complications were not significantly more likely to die (*P* = 0.67).

**Conclusions:**

Single centre and ELSO registry experience provide results of a cohort of children with refractory SA managed with ECLS support. Further study is necessary to determine if use of ECLS in this setting produces better outcomes than careful mechanical ventilation and medical therapy alone.

## Introduction

Asthma is a growing health problem in the USA, affecting over 9 million children under the age of 18 years [1]. Asthma prevalence is at historically high levels, and it remains the most common cause of hospitalisation among children [1], with rates highest among African American children [2,3].

Status asthmaticus (SA) is also a very common indication for admission to the paediatric intensive care unit (PICU). SA is defined as failure of conventional therapy with progression towards respiratory failure due to asthma [4]. SA can progress quickly to a life-threatening emergency in children. Death rates attributable to asthma and SA have been reported at 2.6 per 1 million children annually (186 children) with a significantly higher rate in African American children aged 0 to 17 years of about 9.2 per 1 million [1]. Patients with previous ICU admissions, recurrent hospitalisation and those requiring mechanical ventilatory support have an increased risk of a fatal outcome [5-7].

In addition to the routine administration of continuous nebulised beta-adrenergic agonists with intermittent anticholinergics, corticosteroids and oxygen, adjunctive therapies such as magnesium sulfate, methylxanthines, helium-oxygen mixtures, noninvasive ventilation and intravenous beta-agonists have been employed to avoid respiratory failure and intubation [4]. However, a small number of patients fail to respond to these aggressive treatments and require mechanical ventilation. Up to 20% of children with SA admitted to PICUs [8,9] require intubation, with a subsequent increased risk of death [8,9]. An earlier report found that 10% of patients intubated in a PICU had preceding respiratory or cardiopulmonary arrest [10].

Extracorporeal life support (ECLS) could provide adjunctive pulmonary support for intubated asthmatic patients who remain severely acidotic and hypercarbic in spite of aggressive conventional therapy and unconventional therapies, including inhaled anaesthetics [11]. Although potentially helpful, there has been little experience with ECLS in refractory SA reported. Anecdotal case reports have described its use in adults [12-15] and rarely in children [16]. No extensive case review of ECLS in SA exists in the literature. We have noted increased need for and use of extracorporeal support for children with SA failing aggressive medical and anaesthetic therapy in our PICU, and sought to evaluate our single centre experience with this approach. For comparison, we queried an international ECLS database to evaluate paediatric experience with the use of ECLS in patients with severe SA.

## Materials and methods

We queried the ECLS institutional database at Children's Healthcare of Atlanta at Egleston (Children's at Egleston) for paediatric patients with status asthmaticus (International Classification of Diseases (ICD)-9 code 493.91) receiving extracorporeal support at our institution. Children's at Egleston is a freestanding quaternary referral medical centre with 232 inpatient beds and a 30-bed multidisciplinary (non-cardiac) medical-surgical intensive care unit (ICU). The need for informed consent was waived and institutional review board approval was obtained for collection of deidentified data on demographic characteristics, hospital course before ECLS, ventilatory parameters, arterial blood gas measurements and therapeutic interventions before cannulation. The course of ECLS, complications and outcome were also reviewed.

For comparison with our centre series, we reviewed international experience with ECLS use in children with SA through the Extracorporeal Life Support Organization (ELSO) registry. The ELSO registry is a voluntary database tracking consecutive ECLS patient experience for neonates, children and adults from over 100 centres since 1985 [17]. Following a formal request to review the ELSO registry database, approval was granted from the Protocol Chairman of ELSO Registry Committee. Access to the database was obtained through our centre's membership. The ELSO database was queried for directed review of all patients from age 1 to 17 years with a primary ICD-9 diagnosis (493.91) of SA in respiratory failure receiving ECLS.

At Children's at Egleston, bedside point-of-care devices (iSTAT; Abbott Point of Care Inc., Abbott Park, Illinois, USA) are routinely used for blood gas measurement. With this device, the maximal value reported and displayed for partial pressure of arterial carbon dioxide (PaCO_2_) is 'greater than 130 mmHg'. Therefore, for statistical analysis of these values, the value 130 mmHg was used if a maximal PaCO_2 _value was reported. However, based on simultaneous pH measurements, it is likely that traditional blood gas analyser measurements of PaCO_2 _would have yielded significantly higher values.

Ventilatory support for SA in intubated patients in the Children's at Egleston PICU is typically performed using pressure controlled ventilation (Siemens Servoi; Maquet, Bridgewater, NJ, USA). Mechanical ventilation at our centre is directed at allowing adequate expiratory effort, with relatively short inspiratory time and longer expiratory times, with respiratory rates decreased to allow for improved lung emptying based on auscultation, ventilator graphics and measurement of inspiratory plateau pressures. Lower (but not zero) positive end-expiratory pressures are also utilised. Inhaled anaesthesia is provided using isoflurane or sevoforane at minimum alveolar concentration (MAC) starting at 0.5% up to maximum 1.0%. Choices for individual therapies in severe SA were based on physician discretion.

Statistical analysis of data was performed comparing pre-ECLS variables, complications and outcomes using standard statistical software (Sigma-Stat; Systat Software Inc., Richmond, CA, USA). Analysis was performed comparing patients from Children's at Egleston, all patients identified from the ELSO registry and ELSO registry patients excluding those from Children's at Egleston (who are also captured in the ELSO registry).

## Results

### Experience of Children's at Egleston

From 1986 to 2007, 13 patients received ECLS support for refractory SA and hypercarbic respiratory failure failing treatment with conventional and alternative medical therapy (Table 1). Seven of these patients were cannulated in 2006 and 2007 alone. Median patient age was 10 years (range 1 to 16 years). Patients had a median of 3.5 hospitalisations for asthma (range 1 to 4) prior to ECLS hospitalisation. Three of the 13 (23%) children had been previously intubated for asthma. Of the 13 patients at Children's, 93% chronically received inhaled beta agonists, 85% were on daily inhaled corticosteroids and 62% received leukotriene inhibitors at the time of admission. Ten patients were intubated and transferred from an outside medical centre, and only three were intubated at our facility due to respiratory failure.

**Table 1:** Demographic and clinical variables for patients receiving extracorporeal life support for status asthmaticus at Children's Healthcare of Atlanta at Egleston from 1986 to 2007

Patient	Year ECLS performed	Race	Total ECLS run time (hours)	Ventilator hours prior to ECLS initiation	Serum pH prior to ECLS	Serum PaCO_2 _prior to ECLS (mmHg)	Ventilator hours after ECLS until extubation
1	1994	AA	135	20	7.02	102	288
2	1995	C	184	10	6.83	172	89
3	1996	AA	63	17	6.83	186	44
4	1997	AA	174	34	6.98	151	51
5	2002	C	395	24	6.75	190	383
6	2004	AA	100	9.5	6.94	> 130	72
7	2006	AA	106	32	7.03	101	38.5
8	2006	C	95	20	6.93	> 130	142
9	2006	C	66	14	6.99	106	17.5
10	2007	AA	52	12	7.0	127	46
11	2007	AA	48	12	6.85	> 130	30
12	2007	AA	42	4	6.8	> 130	53
13	2007	AA	70	1	6.78	> 130	40

AA = African-American; C = Caucasian; ECLS = extracorporeal life support; PaCO_2 _= partial pressure of arterial carbon dioxide.

Therapeutic interventions for treatment of SA for each patient are shown in Table 2. Prior to initiation of ECLS, all 13 patients at Children's at Egleston received continuous inhaled beta agonists and anticholinergics, intravenous beta agonist (terbutaline) infusion and intravenous corticosteroids. Additionally, 92% received intravenous ketamine infusion, 77% received helium-oxygen blended in ventilator gases and 69% received intravenous magnesium sulfate. Eight of 13 (62%) children also received inhaled anaesthetic agents inline before cannulation. Three of the 13 (31%) children received continuous intravenous theophylline infusion, but none after 1997.

**Table 2:** Medical and anaesthetic therapies used in 13 patients placed on extracorporeal life support at Children's Healthcare of Atlanta at Egleston between 1986 and 2007

Patient	IV beta agonist	Ketamine	Magnesium sulfate	Helium-oxygen	Theophyline infusion	Inhalational agent
1	Y	Y	N	Y	Y	Y
2	Y	Y	N	N	Y	N
3	Y	Y	N	N	N	Y
4	Y	N	N	Y	Y	Y
5	Y	Y	Y	Y	N	Y
6	Y	Y	Y	Y	N	Y
7	Y	Y	Y	Y	N	N
8	Y	Y	Y	Y	N	Y
9	Y	Y	Y	Y	N	Y
10	Y	Y	Y	N	N	N
11	Y	Y	Y	Y	N	N
12	Y	Y	Y	Y	N	N
13	Y	Y	Y	N	N	Y

Total percentage receiving therapy	100%	92%	69%	69%	23%	62%

Median time from patient intubation to ECLS institution was 14 hours (range 1 to 34 hours). Prior to ECLS cannulation, median patient arterial pH was 6.93 (range 6.78 to 7.03), and median PaCO_2 _was at least 130 mmHg prior to cannulation. Maximum ventilator settings prior to ECLS cannulation included median peak inspiratory pressure (PIP) of 51.5 cmH_2_O (range 40 to 72 cmH_2_O), respiratory rate 12 breaths/minute (range 10 to 25 breaths/minute), peak end expiratory pressure (PEEP) 5 cmH_2_O (range 0 to 15 cmH_2_O) and ventilator mean airway pressure of 19 cmH_2_O (range 9 to 24 cmH_2_O). Four patients experienced cardiorespiratory arrest prior to ECLS. Two of these events occurred at outside hospitals and two events, brief in nature, occurred shortly before ECLS was initiated. No cardiorespiratory arrest episodes occurred during ECLS.

Twelve of 13 patients were cannulated by the venovenous (VV) approach. A single patient underwent the venoarterial (VA) approach due to requiring cardiopulmonary resuscitation during cannulation. Median time spent on ECLS was 95 hours (range 42 to 395 hours). One patient developed pulmonary haemorrhage associated with Stachybotrys chartarum infection and required ECLS for 395 hours. Median time of ventilation after decannulation until extubation was 52 hours (range 18 to 393 hours), and median time to PICU discharge after decannulation was 125 hours (Tables 3 and 4).

**Table 3:** Demographic and clinical data for all ELSO registry patients, ELSO registry patients excluding patients from Children's Healthcare of Atlanta at Egleston, and Children's at Egleston patients alone

	All patients (n = 64) median (range)	ELSO alone (n = 51) median (range)	Children's at Egleston (n = 13) median (range)
Age (years)	10 (1 to 17)	10 (1 to 17)	10 (1 to17)
Hours on ECLS (hours)	93 (31 to 395)	91 (31 to 218)	97.5 (42 to 395)
Survival (%)	60/64 (95%)	47/51 (92%)	13/13 (100%)
PEEP (cmH_2_O)	5 (0 to 20)	5 (0 to 20)	5 (0 to 15)
PIP (cmH_2_O)	44 (23 to 130)	42 (23 to 130)	51 (40 to 80)
MAP (cmH_2_O)	15 (3 to 48)	16 (3 to 48)	19 (9 to 24)
Cardiorespiratory arrest prior to ECLS (%)	9/64 (14%)	5/51 (10%)	4/13 (31%)
pH	6.98 (6.75 to 7.28)	6.98 (6.78 to 7.28)	6.89 (6.75 to 7.03)
PaCO_2 _(mmHg)	122 (61 to 284)	121 (61 to 284)	130 (102 to 186)
Percentage VV cannulation use	86%	82%	92%

No significant differences were noted between group variables.

ECLS = extracorporeal life support; ELSO = Extracorporeal Life Support Organization; MAC = minimum alveolar concentration; PaCO_2 _= partial pressure of arterial carbon dioxide; PEEP = peak end expiratory pressure; PIP = peak inspiratory pressure; VV = venovenous.

**Table 4:** Complications reported for all ELSO registry patients, patients from Children's Healthcare of Atlanta at Egleston alone (CHOA) and ELSO registry patients excluding Children's at Egleston patients (non-CHOA)

Organ system dysfunction	All ELSO patients (n = 64)	CHOA alone (n = 13)	ELSO alone (n = 51)	Number of ELSO non-survivors with complication*	CHOA vs. non-CHOA p value
**CNS**	4 (6%)	2 (14%)	2 (4%)	1	0.153
**Cardiovascular**	21 (33%)	5 (38%)	16 (31%)	3	0.421
**Haemorrhagic**	15 (23%)	7 (54%)	8 (16%)	1	0.001**
**Mechanical**	15 (23%)	4 (31%)	11 (22%)	1	0.276
**Pulmonary**	9 (14%)	3 (23%)	6 (12%)	2	0.123
**Metabolic**	20 (31%)	9 (69%)	11 (22%)	1	0.0001**
**Renal**	12 (18%)	3 (21%)	9 (18%)	2	0.354
**Infectious**	10 (16%)	7 (54%)	3 (6%)	1	0.0001**

All values expressed in number (percentage). * All patients at Children's at Egleston survived. ** Statistically significant.

CHOA = Children's Healthcare of Atlanta at Egleston; CNS = central nervous system; ELSO = Extracorporeal Life Support Organization.

Complications relating to SA and ventilation were common prior to cannulation. Pneumothorax occurred in 2 fo 13 (15%) patients prior to admission to the Children's at Egleston PICU. Of concern, two of 13 (15%) children demonstrated unilateral pupillary dilation prior to cannulation with concern for increased intracranial pressure and cerebral oedema. Neither patient had undergone prior cardiorespiratory arrest or significant hypoxia. Computerised tomography did not reveal intracranial abnormalities in either patient. Only one of these patients had accompanying neurological changes (seizure). Abnormalities had resolved at time of decannulation. Four of thirteen (31%) patients experienced cardiorespiratory arrest prior to ECLS while in the PICU.

### Experience of ELSO registry

Sixty-four patients meeting criteria from the ELSO registry were identified. Of the 64, 13 of these patients were registered from Children's at Egleston; thus analysis was performed on both the total 64 SA patients and on the 51 non-Children's at Egleston ELSO SA patients (Table 3).

A significant increase in reported ECLS cases for SA was found from 2002 to 2007 (42 of 64; 66%) compared with 1986 to 2002 (23 of 64; 34%; *P* < 0.0001). Median age of the 64 ELSO registry patients was 10 years (range 1 to 17 years). Median time from intubation to institution of ECLS was 15 hours (range 1 to 230 hours). Median ventilator settings prior to ECLS cannulation included PIP of 44 cmH_2_O, PEEP of 5 cmH_2_O, ventilator rate of 14 breaths/minute and ventilator mean airway pressure of 15 cmH_2_O.

No differences were seen between ELSO patients and Children's at Egleston patients alone in pre-ECLS variables. High frequency oscillatory ventilation was initiated in 6 of 64 (9%); none of these children were from our facility.

For ELSO registry patients, median serum pH prior to ECLS was 6.96 (range 6.78 to 7.28). Median PaCO_2 _was 123 mmHg (range 70 to 237 mmHg), and partial pressure of arterial oxygen (PaO_2_) was 126 mmHg (range 59 to 636 mmHg). Survival in patients with pre-ECLS PaCO_2 _less than 100 mmHg was no different than in patients with PaCO_2 _greater than 100 mmHg (10/11 vs. 50/53; not statistically significant). No correlation was found between decreased serum pH less than 7.0 at time of cannulation and survival. No patient had significant hypoxaemia (PaO_2 _greater than 50 mmHg) reported at the time of cannulation.

Of 64 ELSO patients, 55 (86%) had VV cannula configuration for ECLS support and 9 (14%) had VA support. One patient was converted from VV to VA support during the ECLS run. Reported ELSO use of VV for cannulation increased over the course of the study period, with 38 of 41 (93%) patients having VV ECLS from 2002 to 2007 compared with 17 of 23 (74%) patients from 1986 to 2001 (p = 0.305). Overall ECLS survival was 60 of 64 (94%) patients, including all 13 from our institution. Median time of ECLS support was 94 hours. All nine VA patients (100%) survived compared with 51 of 55 of VV patients (93%; *P* = 0.78). No statistically significant difference in ECLS variables or outcomes was seen between non-Children's at Egleston ELSO patients and Children's at Egleston patients alone.

Cardiovascular, haemorrhagic and mechanical complications were most commonly reported (Table 4). Cardiovascular problems included hypertension in six patients and vasopressor requirements in 15 children. Four of 64 patients experienced central nervous system complications, including seizures and intracranial haemorrhage. However, the presence of neurological complications was not associated with an increased likelihood of death. Children's at Egleston patients reported significantly higher rates of haemorrhagic (cannula site bleeding), metabolic (hyperglycaemia) and infectious complications when compared with ELSO patients (Table 4).

## Discussion

This report provides, to date, the largest single centre experience and the largest international case series of ECLS use in severe SA. Although it provided accumulated experience with ECLS use in severe SA, certain limitations require discussion. This review is inherently limited in its conclusions because of the retrospective nature of the data available both from our institution and from the ELSO registry. Of particular concern, no specific criteria were used to initiate ECLS. Therefore, it is difficult to conclude from this data specific indications for use of ECLS in SA. Evaluation of intensity of asthma therapy prior to ECLS is impossible to interpret in ELSO registry patients because of the voluntary nature of reporting and the lack of available detailed data on ventilator settings and medical therapy. However, more specific information on medical therapies and ventilator settings in SA patients from our single centre could be helpful in evaluating the timing of initiation of ECLS in this subset.

ELSO experience demonstrates a significant increase in reported use of ECLS for SA and respiratory failure since 1995. This rise could be the result of an increasing number of centres performing ECLS, or increased comfort with use of ECLS in this setting based on experience. However, other factors could be responsible, including higher asthma prevalence, increasing regional incidence or severity of asthma, or overall increasing severity of illness.

National trends in asthma could be impacting ECLS use. Ambulatory visits for children with asthma have continued to increase nationally since 2000 [1]. However, inpatient admission rates are unchanged, suggesting that a higher threshold for hospitalisation for asthma exists, and that hospitalised asthma patients are more severely ill at time of admission [3].

Of note, use of ECLS at our institution represents a significant number (20%) of the reported ELSO cases. This finding could be the result of regional asthma severity, lack of aggressive medical or ventilatory therapies or an institutional tendency to turn to ECLS early in severe SA.

Regional severity of asthma could also have resulted in increased use of ECLS in our institution. Asthma incidence and severity have grown in Georgia. Eleven percent of children in Georgia aged 0 to 17 years have asthma [18], making it a state with one of the highest asthma prevalence rates in the country. Children living in high areas of air pollution have higher baseline asthma severity [19]. Atlanta, the home for a majority of patients in our single centre series, was recently ranked poorly among major USA cities for year-round particle pollution and ozone pollution [20] and for overall livability for atopic individuals [21]. Atlanta is also noted to have a crude paediatric and adult asthma death rate worse than the national average [21]. Racial composition of asthma patients in our centre could also have been a factor in the rise in use of ECLS. Disparities in adverse outcomes such as emergency department visits, hospitalisations and death are substantially higher for African-American children [1]. Nine of 13 (69%) patients in the Children's at Egleston cohort were African-American.

Mortality from asthma is potentially avoidable [3] but increases with the need for mechanical ventilation [8,22]. The majority of our patients (10 or 13 or 77%) were intubated at outlying facilities and received varied therapy before transfer to our institution. This experience agrees with recent studies demonstrating a significantly increased incidence of intubation at community facilities when compared with children's hospitals [23,24]. The decision to intubate an asthmatic should not be made without exhausting all therapeutic options including non-invasive positive pressure ventilation [25]. Review of the experience at our centre suggests that medical therapies (Table 2) were assertively used. Ventilator therapies appeared consistent with accepted approaches reported elsewhere [4], and a median time of 14 hours before ECLS use suggests significant interventions were attempted before turning to ECLS.

Dynamic hyperinflation (DHI) chiefly contributes to increasing mortality in an intubated asthmatic patient [6]. Recommended ventilator strategies in SA and DHI are focused on allowing maximal emptying times through low ventilator rates and allowing spontaneous respiration if possible. It is possible that some ELSO registry patients did not receive optimal ventilator strategies prior to cannulating for ECLS. However, the data shows low median ventilator rates and PEEP values prior to ECLS suggesting that, in general, these approaches were taken. Similarly, multiple adjunctive medical therapies have been used and suggested for severe SA, including theophylline, magnesium sulfate, helium-oxygen (heliox) and inhaled anaesthesia [4]. Although the ELSO registry does not provide detailed data on therapy, our institutional data is able to demonstrate aggressive and broad-based medical therapies attempted before resorting to ECLS cannulation.

ECLS in an asthmatic patient allows for lung rest, providing time for bronchiolar relaxation, aggressive pulmonary toilet and even controlled bronchoscopy if needed for plastic bronchitis [26,27]. ECLS cannulation may be performed either with VA or VV cannulation techniques. VV ECLS offers advantages of preserved pulmonary blood flow, preservation of the carotid artery, improved oxygenation of the myocardium, physiological left ventricular cardiac output providing pulsatile blood flow and preservation of normal cerebral blood flow velocities [28]. VA cannulation has the added risks of carotid ligation, cardiac stun and increased cardiac afterload, which can be minimised with VV ECLS. Use of VV support has generally increased relative to VA over the past decade for respiratory failure ECLS [28]. VV support is likely to be the best choice for an asthmatic patient given the relatively low blood flows required to remove plasma carbon dioxide and lack of need for cardiac support. Other devices can provide extracorporeal carbon dioxide removal in a pumpless fashion [29].

Mortality in SA secondary to air leak syndromes and cerebral oedema may be unavoidable with most conventional medical and ventilatory therapies.

Although permissive hypercapnoea has become an important strategy in ventilating asthmatic patients [4,30,31], no consensus exists regarding acceptable levels of hypercapnoea. Several case reports describe diffuse cerebral oedema, subarachnoid haemorrhage, quadriparesis, hyperreflexia and extensor plantar reflexes associated with severe hypercarbia in SA [32-36]. In one report, an 11-year-old patient with asthma developed subarachnoid haemorrhage thought to be secondary to hypercarbia with a maximum PaCO_2 _of 135 mmHg [32]. CNS complications associated with hypercarbia are likely to be due to dilation of cerebral vasculature and marked increases in cerebral blood flow [37]. Blood flow changes coupled with decreased venous return secondary to increased intrathoracic pressure and prolonged acidosis may produce cerebral oedema, stroke and even death in patients with asthma [32,34,35]. Elevated tau protein, associated with neuronal cell death, has been reported in the cerebrospinal fluid of an asthmatic patient with hypercarbia [38].

Central nervous system complications occurred in four ELSO registry patients. Of note, these were not associated with prior cardiorespiratory arrest or survival. However, it is impossible to ascertain the timing of either injury or arrest relative to ECLS initiation from the registry, or to be able to speculate whether earlier use of ECLS would have prevented cardiopulmonary arrest in registry patients. Hypoxia and anoxic injury would likely be a significant contributor to morbidity and mortality in SA, but it is not possible to determine if these patients with central nervous system complications had significant hypoxic injury or complications before admission or arrest. In the experience of Children's at Egleston, two of four patients undergoing cardiorespiratory arrest had their events at outlying hospitals but were able to be cannulated without requiring VA support for cardiac dysfunction. The retrospective nature of the ELSO registry and centre experience limits the ability to determine outcome of these SA patients in the absence of ECLS.

## Conclusions

Collective ELSO and single centre experience describes the use of ECLS as an adjunctive therapy for children with severe refractory SA. VV cannulation methods provided adequate support in this setting.

Given its high costs and potential complications, however, further study is indicated to determine if the use of ECLS provides outcome benefits over careful mechanical ventilation and medical therapies alone.

## Key messages

• The ELSO registry provides accumulated experience with the use of ECLS in refractory SA and respiratory failure.

• ECLS can be effectively provided for SA with VV cannulation methods.

• Further study is necessary to determine efficacy and timing of ECLS in severe SA compared with standard therapies alone.

## Abbreviations

DHI: dynamic hyperinflation; ECLS: extracorporeal life support; ELSO: Extracorporeal Life Support Organization; ICD: International Classification of Diseases; ICU: intensive care unit; MAC: minimum alveolar concentration; PaCO_2_: partial pressure of arterial carbon dioxide; PaO_2_: partial pressure of arterial oxygen; PEEP: peak end expiratory pressure; PICU: paediatric intensive care unit; PIP: peak inspiratory pressure; SA: status asthmaticus; VA: venoarterial; VV: venovenous.

## Competing interests

The authors declare that they have no competing interests.

## Authors' contributions

MH and WCP gathered organised data. KH analysed and compared the data and wrote the manuscript with the assistance of TP and JDF.
